# Major complications after intraoperative radiotherapy with low-energy x-rays in early breast cancer

**DOI:** 10.1007/s00066-023-02128-z

**Published:** 2023-08-17

**Authors:** Laura Berger, Anja Grimm, Marc Sütterlin, Saskia Spaich, Elena Sperk, Benjamin Tuschy, Sebastian Berlit

**Affiliations:** 1 Department of Obstetrics and Gynecology, University Medical Center Mannheim, Heidelberg University, Theodor-Kutzer Ufer 1-3, 68167 Mannheim, Germany; 2 Department of Radiation Oncology, University Medical Center Mannheim, Heidelberg University, Mannheim, Germany

**Keywords:** Breast-conserving surgery, Partial breast irradiation, Intrabeam, Complication, Wound infection

## Abstract

**Purpose:**

To describe and analyze major local complications after intraoperative radiotherapy (IORT) with low-energy x‑rays during breast-conserving surgery (BCS) in early breast cancer.

**Methods:**

Ten women out of 408 who were treated with IORT between 2002 and 2017 and subsequently developed a severe local complication requiring surgical intervention were retrospectively identified and analyzed. Demographic, clinical, and surgical parameters as well as complication characteristics and treatment methods were evaluated.

**Results:**

At initial presentation, eight patients (80%) showed redness, six (60%) seroma, six (60%) wound infection, six (60%) suture dehiscence, and four (40%) induration of the former surgical area. Hematoma and necrosis were observed in one case (10%) each. Time interval until appearance of the first symptoms ranged from directly postoperative until 15 years postoperatively (median 3.1 months). Initial treatment modalities comprised antibiotic therapy (*n* = 8/80%) and transcutaneous aspiration of seroma (*n* = 3/30%). In the majority of patients, smaller surgical interventions (excision of a necrotic area/fistula [*n* = 6/60%] or secondary suture [*n* = 5/50%]) were sufficient to overcome the complication, yet larger interventions such as complex flap surgery and mastectomy were necessary in one patient each.

**Conclusion:**

IORT is an efficient and safe treatment method as < 2.5% of all IORT patients experienced major local complications. However, it seems to pose the risk of causing severe local complications that may require lengthy and burdensome treatment. Thorough preoperative counseling, implementation of recommended intraoperative precautions, and high vigilance for first symptoms of complications during follow-up appointments are necessary measures.

## Introduction

With an estimated 2.3 million new cases and 685,000 deaths in 2020, breast cancer is the most frequent cancer in women worldwide [1]. Diagnosis in early stages is the key to effective and successful treatment with limited side effects. The usual locoregional therapeutic approach for early breast cancer consists of breast-conserving surgery (BCS) with axillary sentinel lymph node biopsy (SNLB) followed by postoperative whole-breast radiotherapy (WBRT). Systemic treatment comprises, depending on tumor and patient characteristics, chemotherapy, endocrine therapy, and targeted agents. Postoperative WBRT to eradicate clinically occult tumor cells markedly reduces the risk of local recurrence, which also translates into a reduction of mortality [2, 3]. In patients showing risk factors for local recurrence, delivering an additional radiation dose (boost) to the tumor bed further decreases the local recurrence rate [4, 5]. As the majority of ipsilateral recurrences occur within close proximity to the original site of disease [6, 7], the concept of partial-breast irradiation (PBI) as a more risk-adapted radiotherapy strategy has evolved. It can be achieved by various techniques that have been tested in large-scale clinical trials such as intracavitary (Mammosite®, Hologic, Inc., Marlborough, Massachusetts, USA) or interstitial brachytherapy, external beam radiotherapy (EBRT), or intraoperative radiotherapy (IORT) with low-energy x‑rays or electrons (IOERT) [8–12]. Compared to other methods, IORT enables immediate und precise delivery of radiotherapy during BCS, thus avoiding a temporal and geographic miss. Thereby, the time for repopulation of possible residual tumor cells is minimized and accurate dose delivery to the target tissue despite increasingly complex oncoplastic reconstruction techniques is made possible [13]. The recently published long-term results of the randomized non-inferiority phase III TARGIT‑A trial showed that IORT using low-energy x‑rays is an effective alternative to EBRT for selected early breast cancer patients with comparable long-term cancer control, as there were no statistically significant differences regarding local recurrence-free survival and overall survival between the study and the control group. Notably, non-breast cancer mortality was reduced in the IORT group [12]. In clinical routine, the process of patient selection requires particular attention and consists of a two-step procedure: preoperatively, topographical, histological, and biological tumor features are assessed (inclusion criteria are, among others, age > 50 years, tumor size ≤ 3 cm, unifocal and unicentric localization, no lymph vessel invasion [L0], and no hemangiosis [V0]). The final decision on whether IORT can be performed is made during the subsequent intraoperative phase based on histological examination of frozen sections, freedom of margins, and negative status of sentinel nodes [14]. Besides oncological safety, cosmetic outcomes and acute as well as chronic toxicities are of increasing relevance, especially when comparing treatment options with similar efficacy and survival rates. The most frequent local side effects caused by radiotherapy of the breast include fibrosis, telangiectasia, edema, erythema, hyperpigmentation, ulceration, skin retraction, pneumonitis, pain, seroma, and fat necrosis [15]. Overall, the complication rate with IORT appears to be relatively limited and is confined to low-grade events in the majority of cases: any complication in 17.6% of the patients, major toxicity in 3.3% of the patients (defined as skin breakdown or delayed wound healing and Radiation Therapy Oncology Group [RTOG] toxicity grade of 3 or 4) [16]. However, in rare cases, we observed severe and long-lasting local complications which lead to additional surgical interventions and required intensive and long medical wound care. In the literature, there are only few comments on major local complications after IORT and even more so on details regarding therapy and risk factors. Thus, the purpose of this study was to evaluate the patient and treatment characteristics of women who suffered severe local complications after IORT and to evaluate those complications from their first presentation to their successful treatment.

## Materials and methods

This study is a retrospective case series and was approved by the Ethics Committee II of the University of Heidelberg, Medical Faculty Mannheim (2018-501N-MA). Among all women (*n* = 408) with early breast cancer who underwent BCS with IORT at University Medical Center Mannheim between January 2002 and October 2017, ten women who were subsequently treated due to major complications requiring surgical intervention were identified by retrospective patient chart review. The patient data including demographic parameters, tumor characteristics, treatment and surgery details, as well as complication-related information, were obtained from the patient chart.

IORT was performed during BCS using the mobile Intrabeam® device, a low-energy x‑ray system operating at a peak voltage of 50 kV (Carl Zeiss Meditec, Oberkochen, Germany). After tumor removal, the appropriate spherical applicator (sizes ranging from 1.5 to 5.0 cm) was placed in the surgical cavity and a single dose of 20 Gy (prescribed to the applicator surface) was delivered to the surface of the tumor bed. Additional WBRT, if necessary, was performed according to national guidelines with a total dose of 46–50 Gy using conventional fractionation. Systemic therapy (neoadjuvant or adjuvant chemotherapy, targeted therapy as well as endocrine therapy) was carried out in accordance with national guidelines at the respective time of treatment and individual recommendations of the interdisciplinary oncological board.

All data were collected in a Microsoft Excel spreadsheet. Quantitative data are presented as median and range; for qualitative data, absolute and relative frequencies are given.

## Results

### Demographic parameters

The median age at the time of breast surgery was 60 years and median body mass index (BMI) was 26.4 kg/m^2^. Two patients (20%) had a history of contralateral breast cancer. Previous ipsilateral and contralateral breast surgery due to benign tumors was documented in one case each. Further demographic parameters are shown in Table 1.

**Table 1:** Demographic characteristics

Variable	Median and range/frequency
*Age at primary surgery (years)*	60.0 (49–82)
*BMI (kg/m*^*2*^*)*	26.4 (20.1–40.9)
*Smoking status*	
Yes	2 (20%)
No	7 (70%)
Formerly smoking	1 (10%)
*Alcohol consumption*	
Yes	3 (30%)
No	7 (70%)
*Chronic diseases*	
Hypertension	5 (50%)
Hypercholesterolemia	2 (20%)
Bronchial asthma	1 (10%)
Iron deficiency anemia	1 (10%)
Thrombocytosis	1 (10%)
Polyneuropathy	1 (10%)
History of stroke	1 (10%)
History of ovarian cancer	1 (10%)
*Previous breast surgery*	
Ipsilateral	1 (10%)
Contralateral	1 (10%)

*BMI* Body mass index

### Tumor and treatment characteristics

Tumor characteristics such as localization, tumor stage, and histopathological features are summarized in Table 2. Half of the women received chemotherapy: in two of the cases (20%) it was applied in a neoadjuvant setting and in three cases (30%) in an adjuvant setting. WBRT was completed in seven of the cases (70%). Two patients (20%) did not receive WBRT (even though it was indicated due to unfavorable tumor characteristics): in one case, radiation was refused due to impaired wound healing and in the other case, the reason remained unclear. One patient stopped radiation after receiving 38 Gy due to occurrence of suture dehiscence. Regarding non-surgical treatment methods, three patients were treated with both chemotherapy and radiation and four patients received radiation only but did not require chemotherapy. The patient who stopped radiation due to suture dehiscence had undergone neoadjuvant chemotherapy. Out of the two patients who did not receive radiation, one was treated with adjuvant chemotherapy and the other did not require chemotherapy.

**Table 2:** Tumor and treatment characteristics

Variable	Frequency (%)
**Affected side**	
Right	6 (60)
Left	4 (40)
**Affected quadrant**	
Upper outer	8 (80)
Lower outer	1 (10)
Central	1 (10)
**Tumor stage**	
cT1a	0 (0)
cT1b	3 (30)
cT1c	5 (50)
cT2	2 (20)
**Histopathologic subtype**	
NST	7 (70)
ILC	2 (20)
Atypical medullary carcinoma	1 (10)
**Grading**	
G1	1 (10)
G2	5 (50)
G3	4 (40)
**Receptor status**	
ER+/PR+/HER2-	5 (50)
ER+/PR-/HER2-	1 (10)
ER+/PR+/HER2+	1 (10)
ER-/PR-/HER2-	3 (30)
**Chemotherapy**	
*No*	5 (50)
*Yes*	5 (50)
Neoadjuvant	2 (20)
Adjuvant	3 (30)
**WBRT**	
*Yes*	7 (70)
*No*	2 (20)
Refused due to impaired healing	1 (10)
Reason not clear	1 (10)
*Stopped*	1 (10 )

*NST* carcinoma of no special type, *ILC* invasive lobular carcinoma, *ER* estrogen receptor, *PR* progesterone receptor, *HER2* human epidermal growth factor receptor 2, *WBRT* whole-breast radiotherapy

### Surgical parameters (initial resection of the tumor)

Median duration of initial surgery for primary tumor resection with subsequent IORT was 138 min. Four of the patients (40%) received axillary lymph node dissection (ALND) and six of the patients (60%) sentinel lymph node biopsy (SLNB). The largest IORT applicator (5 cm in diameter) was used in half of the cases. The 4.5- and 3.5-cm applicators were used in two cases each and the 3.0-cm applicator was used once. Further surgical and perioperative parameters are depicted in Table 3.

**Table 3:** Surgery characteristics

Variable	Median and range/frequency
*Duration of surgery (min)*	138 (105–208)
*Preoperative wire marking*	
Yes	8 (80%)
No	2 (20%)
*Axillary surgery*	
SLNB	6 (60%)
ALND	4 (40%)
*Skin incision type*	
Semicircular	8 (80%)
Radial	0 (0%)
Unknown	2 (20%)
*Overlying skin excised*	3 (30%)
*Weight of main specimen (g; n* *=* *5)*	20 (12–38)
*Volume of main specimen (cm*^*3*^*)*	63.1 (15.3–200)
*Sonographic tumor-skin distance (mm; n* *=* *5)*	12 (9–20)
*Applicator size*	
5 cm	5 (50%)
4.5 cm	2 (20%)
4 cm	0 (0%)
3.5 cm	2 (20%)
3 cm	1 (10%)
*Oncoplastic surgery*	
Yes	6 (60%)
No	3 (30%)
Unknown	1 (10%)
*Breast wound drain*	9 (90%)
*Duration of breast drainage (d)*	3 (1–8)
*Overall breast drainage output (ml)*	80 (5–700)
*Axillary wound drain*	5 (50%)
*Duration of axilla drainage (d)*	2 (1–3)
*Overall axilla drainage output (ml)*	20 (10–70)
*Postoperative antibiotic prophylaxis with oral cefuroxime for 3 days*	
Yes	8 (80%)
No	2 (20%)
*Duration of hospital stay (d)*	6 (3–12)

*SLNB* sentinel lymph node biopsy, *ALND* axillary lymph node dissection

### Management of complications

Complication characteristics are presented in Table 4. The median time interval until first symptoms of local complications occurred was 3.1 months, but showed a wide range from directly postoperative up to 15 years postoperatively. The median follow-up time after first symptoms of the complication was 58.5 months (1–123 months). Regarding the temporal relation between the first occurrence of the complication and realization of WBRT, four patients (40%) presented with first symptoms of the complication after completion of WBRT and one patient (10%) during the course of WBRT. In five patients (50%), the complication became clinically evident before the (intended) start of WBRT. It has to be noted that the two patients who did not receive WBRT are included in this group: in one patient, the complication manifested directly after surgery and WBRT was refused due to a non-healing wound, and in the other patient, the reason for not undergoing WBRT after surgery remained unclear and the complication occurred 14 years after surgery with IORT treatment. In the patients who received WBRT before the complication became evident or developed the complication during the course of WBRT, the median time interval between surgery (including IORT) and start of WBRT was 50 days (29–290 days). Concerning the temporal relation between the first occurrence of the complication and chemotherapeutic treatment, the two patients with neoadjuvant chemotherapy and two of the three patients with adjuvant chemotherapy developed the complication after completion of chemotherapy. One patient had the complication during adjuvant chemotherapy. Frequent symptoms and findings at first presentation were redness (*n* = 8/80%), seroma (*n* = 6/60%), local wound infection (*n* = 6/60%; either confirmed by swab culture and/or diagnosed by the physician based on the clinical manifestation), suture dehiscence (*n* = 6/60%), and induration (*n* = 4/40%). Hematoma and necrosis appeared in one of the cases each (10%), none of the patients had fever. Initial treatment modalities were antibiotic therapy administered for more than 3 days in six cases (60%) and puncture of the seroma in three cases (30%). Important in this context is the fact that seroma puncture was only assessed as a therapeutic measure after wound infection had already been diagnosed (of the six patients with wound infection, two underwent seroma puncture subsequently; the third patient from our collective who underwent puncture of the seroma did not have a wound infection). Regarding initial antibiotic therapy, three patients (30%) received cefuroxime and one patient each was treated with amoxicillin/clavulanic acid, ciprofloxacin, or flucloxacillin, respectively. In two patients the antibiotic regimen was switched during the course of treatment due to poor wound healing progress and detection of further bacteria in repeated swab cultures (one patient first received flucloxacillin followed by clindamycin and then moxifloxacin, another patient first had ciprofloxacin, then cotrimoxazole, and finally cefuroxime). Surgical intervention was performed in all of the patients of the presented study collective. While six patients underwent complication-related surgery once, one patient had two interventions, another patient had three interventions, yet another four interventions, and one patient even had to undergo surgery nine times. Regarding the type of surgical intervention, excision of a necrotic area or fistula (*n* = 6/60%) or secondary suture (*n* = 5/50%) were performed most frequently. In five patients, excision of a necrotic area/fistula was performed once, in one patient three times. Concerning secondary suture, two patients had to undergo this intervention once, two patients twice, and one patient three times. One patient (10%) received negative-pressure wound therapy (NPWT) with multiple changes of the dressing and had to undergo complex flap surgery in the further course from a plastic surgeon. Another patient (10%) chose to have a mastectomy to overcome refractory wound complications.

**Table 4:** Complication characteristics

Variable	Median and range/frequency
*Time interval until first symptom of complication*	3.1 months (1 day–15 years)
*Temporal relation of complication and WBRT*	
Before (intended) start of WBRT	5 (50%)
During WBRT	1 (10%)
After completion of WBRT	4 (40%)
*Symptoms at first presentation*^a^	
Redness	8 (80%)
Seroma	6 (60%)
Wound infection	6 (60%)
Suture dehiscence	6 (60%)
Induration	4 (40%)
Hematoma	1 (10%)
Necrosis	1 (10%)
Fever > 38.5 °C	0 (0%)
*Antibiotic therapy >* *3 days*	6 (60%)
Cefuroxime	3 (30%)
Amoxicillin/clavulanic acid	1 (10%)
Ciprofloxacin	1 (10%)
Flucloxacillin	1 (10%)
*Puncture of seroma*	3 (30%)
*Surgical intervention*	10 (100%)
*Number of inventions*	1 (1–9)
*Type of surgical intervention*^a^	
Excision of necrosis or fistula	6 (60%)
Secondary suture	5 (50%)
NPWT	1 (10%)
Mastectomy	1 (10%)
Flap surgery	1 (10%)
Referral to plastic surgery	2 (20%)

*WBRT* whole breast radiotherapy, *NPWT* negative-pressure wound therapy

^a^Multiple answers possible

In two patients a surgical intervention was performed promptly after the complication occurred; one patient with suture dehiscence in the absence of a wound infection 3 weeks after primary surgery received secondary suture, the other patient presenting 14 years after primary surgery with a fistula and recurrent discharge of serous fluid underwent excision of the fistula and secondary suture. The other patients were first treated with non-surgical methods (either in combination or alone) such as antibiotic therapy, puncture of seroma, or medical wound care (e.g., wound irrigations or treatment with antimicrobial dressings or hydrocellular gel dressings). However, as the clinical condition worsened, surgical intervention became necessary. The treatment process often required regular visits over a long period of time (duration ranging from several weeks to over a year).

**Fig. 1:**
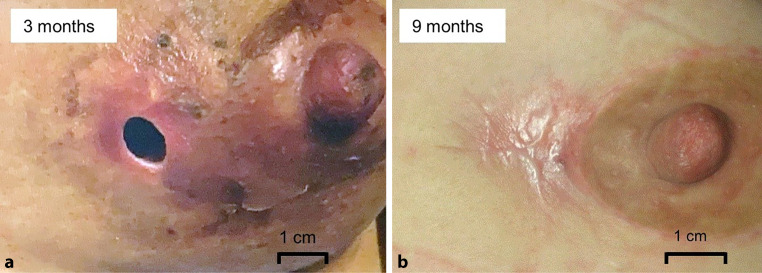
**a** Wound infection with necrosis and concomitant suture dehiscence 3 months postoperatively in a patient who underwent intraoperative radiotherapy during breast-conserving surgery with axillary lymph node dissection. The complication first occurred during the course of whole-breast radiotherapy. **b** After a multimodal therapeutic approach with medical wound care, antibiotic therapy, and three surgical interventions (1 × excision of necrosis, 2 × secondary suture), the wound is healed 9 months postoperatively

A representative case of a major local complication treated with a multimodal regimen is depicted in Fig. 1.

## Discussion

On the whole, IORT is an efficient and safe treatment method. Advantages of IORT include a shortening of overall treatment time, a continuously high quality of life, and a faster resumption of work and daily activities [17–19]. There is no evidence of acute or chronic cardiac toxicity after IORT [20, 21]. However, IORT seems to pose the risk of causing severe local complications that could only be resolved by additional surgical intervention. Further initial treatment modalities were > 3-day course of antibiotic therapy or puncture of seroma. In single cases, in order to control the situation long term, invasive, disfiguring, and straining procedures such as mastectomy or complex flap surgery were necessary. This is the first report of a compilation of major complications that occurred after IORT with low-energy x‑ray during BCS with an analysis of the treatment methods applied to manage them lastingly.

### Toxicity rates after IORT in BCS

In previous studies, short-term complications and acute toxicity within the first postoperative week after IORT were reported to be rare and no grade 3/4 toxicities were observed [22, 23]. A retrospective analysis by Tuschy et al. of 208 patients treated with IORT revealed that suggillation (24%) and palpable seroma (17.3%) were the most frequent postoperative side effects. Furthermore, 13% of the patients showed erythema grade 1–2 and 3.4% had mastitis. In 1.4% of the cases, a surgical revision due to a hematoma or insufficient postoperative hemostasis was necessary [23]. Kraus-Tiefenbacher et al. reported in their prospective study overall lower acute toxicities 1 week after surgery in the IORT group compared to the results by Tuschy et al. [22]: erythema grade 1–2 in 3% of the IORT patients (0% in the BCS-only patients), mastitis in 2% of the IORT patients (compared to none in the BCS-only group), delayed wound healing in 2% (compared to 6%), and hematoseroma in 6% (8% in the BCS-only group). During follow-up at 1, 2, and 4–6 months after surgery there were no cases of delayed wound healing. In a retrospective analysis of patients who received IORT as a tumor bed boost by Stoian et al., only grade 1 toxicities were observed postoperatively. The most frequent event was seroma/hematoma of the breast in 10.3% of the patients; all other toxicities such as dermatitis, wound infection, wound dehiscence, or seroma/hematoma of the axilla each occurred in under 2% of the patients [24]. A study including 93 patients by Gülcelik et al. assessing wound complications within the first month after IORT reported considerably higher rates of complications: seromas were observed in 25.5% of patients in the IORT group (compared to 6% in the BCS-only group), surgical site infections in 21% of the IORT patients (2% in the BCS-only patients), and delayed wound healing in 35% (compared to 8%). There was no hematoma in the IORT group (2% in the BCS-only group). 7% of the patients showed a minor wound dehiscence in the IORT group, while none was observed in the BCS-only group. It was concluded that IORT could have a negative effect on seroma formation, surgical site infection, and wound healing and, hence, the adverse effects of IORT on wound complications should be closely monitored [25].

Regarding long-term toxicities, a study by Sperk et al. reported that patients treated with IORT alone had about half the risk for developing higher-grade toxicities as compared to standard whole-breast radiotherapy (hazard ratio 0.46 [95% CI 0.26–0.83], *p* = 0.010). At 3 years, there were significantly fewer telangiectasias in the IORT group (either IORT alone or IORT + WBRT for risk factors) compared to the WBRT group. Regarding higher-grade fibrosis, only 5.9% of the patients with IORT alone were affected, whereas 37.5% of the patients treated with IORT + WBRT and 18.4% in the WBRT group were affected. All other higher-grade toxicities such as edema, retraction, ulceration, lymphedema of the arm, hyperpigmentation, or pain were similar in both. An interval of less than 5 weeks between IORT and the beginning of WBRT was identified as a predictor for higher grade fibrosis, whereas applicator size or dose rate did not exert a significant impact [26]. In line with this finding, Wenz et al. observed a statistically significant tendency for increased late toxicity when EBRT was initiated early after IORT and therefore suggested not starting EBRT until 5–6 weeks after IORT to reduce the number of toxicity events [27]. Regarding late toxicities at 3 years after IORT as a single modality treatment or boost, Key et al. reported that grade 2 or higher breast toxicity occurred significantly more often in women treated with IORT + WBRT than with IORT alone (2.4 vs. 46.6%; *p* < 0.0001), such as significantly more cases of higher-grade fibrosis (2.4 vs. 43.3%; *p* < 0.0001) and higher-grade retraction (0 vs. 23.3%, *p* = 0.002) in the IORT + WBRT group. In our cohort, all patients had an indication for subsequent WBRT due to unfavorable tumor characteristics (however, only seven patients [70%] completed WBRT: one patient refused and one patient stopped WBRT, in one patient the reason for not undergoing WBRT remained retrospectively unclear). Regarding the time relation between WBRT treatment and occurrence of the complication, we observed a similar number of patients who completed WBRT before the complication became evident and of those who presented with the complication before beginning of WBRT (before start of WBRT: 50% [*n* = 5]; after completion of WBRT: 40% [*n* = 4]; during WBRT: 10% [*n* = 1]).

Hence, upon reviewing the literature, overall toxicity rates concerning IORT in patients undergoing BCS are low. However, due to a potential negative impact on short-term complications, there should be a close focus on wound healing and seroma formation within the first month after surgery, especially in patients undergoing subsequent WBRT. WBRT should not be initiated within the first 4 weeks after BCS with IORT.

### Risk factors for perioperative complications

Compared to the number of studies investigating the oncological efficacy and analyzing toxicities after IORT, studies identifying risk factors for complications after IORT are scarce.

Both the studies by Wang et al. and Rakhra et al. reported that the size of the applicator was significantly associated with an increase in wound complications. However, it remained unclear whether this effect was due to larger incisions or due to the dose distribution associated with a larger applicator [28, 29]. Compared to these studies, the average size of the applicator used in our cohort was even greater, as in 50% of our patients the largest applicator (5.0 cm) was used. A study by Moradi et al. applying a Monte Carlo simulation method to calculate the dose received by the breast skin depending on the applicator size and on the distance between skin and applicator revealed a considerable risk of overdosing the skin. Use of larger applicators was shown to generally result in higher skin doses. For example, using the 5‑cm applicator at a treatment depth of 0.5 cm resulted in delivering a dose as high as 12 Gy to the skin (whereas 6 Gy is rated as the dose limit for the epidermis layer in order to avoid transient skin injury). With all simulated applicator sizes (besides the smallest applicator of 1.5 cm diameter), a distance of 0.5 cm resulted in doses above 6 Gy being delivered to the skin. Increasing the distance to 1.0 cm, the three largest applicators (4.0, 4.5, and 5.0 cm) still exceeded the 6‑Gy threshold. These calculations suggest that the recommended skin-to-applicator distance of 0.5–1.0 cm does not guarantee skin safety, especially when using large applicators [30].

In our study, data on the sonographic distance between tumor and skin was available for only five patients. Median distance was 12 mm (range 9–20 mm). However, since a small rim of tissue surrounding the tumor is taken out in order to obtain clear margins, after tumor removal, the actual distance between applicator and skin is likely to be even shorter. In our study, the combination of using relatively large applicators and having a relatively short tumor–skin distance might serve as a partial explanation for the development of major local complications.

Analyzing early wound fluid production collected in intraoperatively placed suction drains, Ebner et al. observed that both the IORT (*n* = 99) and the non-IORT group (*n* = 53) had a median wound fluid production of 50 ml in the breast (IORT group: range 15–304 ml; non-IORT group: range 2–343 ml). Axillary suction drains produced 49 ml in the IORT group (range 10–240 ml) and 40 ml in the non-IORT group (range 10–285 ml). Overall, wound fluid production in both breast and axilla did not show significant differences. Furthermore, there was no significant difference regarding the median number of days until removal of breast and axilla drains (breast drains: median of 3 days in both IORT and non-IORT patients: IORT patients range 1–6 days, non-IORT patients range 2–6 days; and axilla drains: median of 3 days in both IORT and non-IORT patients: IORT patients range 1–4 days, non-IORT patients range 2–6 days). It was shown that wound fluid production and time until drain removal in both IORT and non-IORT patients did not depend on tumor size [31]. These observations are well in line with the drainage handling in our collective: median overall wound fluid production in the breast was 70 ml (5–700 ml) and median number of days until removal of the breast drain was 3 days (range 1–8 days). In the axilla, we observed a median wound fluid production of 20 ml (10–70 ml) and median number of 1.5 days (1–3 days) until removal of the axilla drain. There are presumably no clinically relevant differences concerning early wound fluid drain in patients with and without IORT. As an in-house policy, suction drain removal in our collective was accomplished when 30 ml or less wound fluid were collected within 24 h. It is debatable to what extent a longer wound fluid drainage would impact seroma formation.

Joseph et al. observed that implementation of postoperative antibiotic prophylaxis led to a decrease in wound infections from 25 to 11% in patients treated with IORT [32]. Another study by Zur et al. showed that patients receiving prophylactic antibiotics had a significantly reduced risk of a wound infection (15.2% vs. 6.6%, OR 0.39, 95% CI 0.2–0.78, *p* = 0.007) [33]. In our study population, the majority of patients (80%) received postoperative antibiotic prophylaxis for 3 days. Hence, administration of prophylactic antibiotic treatment substantially reduces the number of wound infections but does not prevent them completely. Therefore, antibiotic prophylaxis should be applied routinely.

In our cohort, 50% of the patients underwent chemotherapy. Other studies analyzing similar populations in terms of oncological risk profile and thus the indication for subsequent WBRT reported slightly lower rates of chemotherapy (Blank et al. 41%, Vaidya et al. 39%) [34, 35]. It has to be mentioned that conservative oncology is constantly evolving and the indications for chemotherapy are changing over time. Chemotherapeutics have been shown to block the pathways responsible for effective wound repair by inhibition of cell metabolism, cell division, and angiogenesis. Additionally, production of collagen is reduced due to restrained fibroblast proliferation and the risk of wound infections is elevated as a result of the impaired immune system [36]. However, adjuvant chemotherapy was shown to have no impact on chronic higher-grade toxicities in patients treated with IORT and WBRT [37, 38]. Moreover, a retrospective analysis of patients who received neoadjuvant chemotherapy and subsequently underwent IORT and EBRT did not reveal any severe acute and late toxicity [39]. In one of the patients in our study population, the complication became evident during the course of adjuvant chemotherapy, suggesting that all different components of the multimodal therapy strategy in early breast cancer can contribute to the development of complications.

Interestingly, none of the patients in our cohort suffered from diabetes. Overall, diabetes patients have a greater risk of poor wound healing due to chronic inflammation, impaired angiogenesis, macro- and microvascular dysfunction, hyperglycemia, and hypoxia. Regarding breast surgery, it was shown that diabetes is a risk factor for wound healing complications among patients undergoing postmastectomy implant-based reconstruction [40] as well as cosmetic augmentation mastopexy [41]. Zur et al. reported that the presence of diabetes mellitus type II was associated with an increased risk for wound dehiscence in patients treated with IORT [33].

According to the review of literature, increasing applicator size, small tumor–skin distance, omission of prophylactic antibiotic therapy, and diabetes mellitus have been identified as risk factors for short-term toxicity when undergoing IORT in BCS.

### Duration until occurrence of complications

In our cohort there was a remarkably wide range regarding the time interval between the date of treatment with IORT and first presentation of the observed complication, ranging from directly postoperative until 15 years after surgery (median 3.1 months). Only very few studies provide information concerning the duration of this time interval, as cumulative incidences of the complications are usually reported and patients are examined at prescheduled follow-up appointments. To our knowledge, follow-up time in other publications is shorter than in our cohort [16, 28, 29]. In a prospective study on short-term complications within the first year after IORT including 395 patients, Zur et al. reported that wound infections occurred after a median time of 28 days (range 3–316 days) and seroma after a median time of 44 days (range 0–264 days). Median time to appearance of wound dehiscence was 25 days (range 0–290 days) [33]. Concerning physiological wound healing capacities, Zhou et al. found that the average time for the skin incision after BCS to heal was longer in the IORT group as compared to the BCS-only group (13–22 days vs. 9–14 days) [42]. In accordance with the literature discussed above, the first 4 weeks after accomplished IORT seem to be the particularly crucial time period for short-term complications.

Although of arguable clinical relevance, IORT leads to distinct radiological findings reflecting the frequently observed persistent wound cavities filled with hematoma/seroma in which fat necroses develop over time [43–45]. Mammographic and sonographic long-term follow-up (> 3 years) revealed that patients treated with IORT have a significantly higher incidence of fat necroses/oil cysts as compared to those treated with WBRT (57% vs. 17%, *p* = 0.0004) and a significantly higher rate of hematoma/seroma (76% vs. 37%, *p* < 0.0001). In each case, the median size of the abovementioned alterations was higher in the IORT group [43]. Furthermore, in mammographic follow-ups, patients treated with IORT exhibited scar calcifications more frequently (63% vs. 19%, *p* = 0.002) [45]. Whereas patients who underwent WBRT only showed minimal mammographic and sonographic alterations of the tissue 24 months after treatment, in patients treated with IORT, these alterations became progressively more evident during the course of a 24-month follow-up [46]. These findings imply that treatment with IORT causes extensive and persisting alterations within the breast parenchyma that continue to exist after the wound healing process seems completed from an external point of view. To what extent these circumstances explain the occurrence of a major complication as late as 15 years after IORT as depicted above is debatable.

### Handling of complications

Zur et al. reported in their study on short-term complications after IORT that 3.3% of the patients (*n* = 13) required hospitalization due to an infection, eight of them needed surgical drainage. Wound dehiscence occurred in 8.1% of the patients (*n* = 32), two patients (0.5%) required secondary suture. Furthermore, there were two small skin necroses (< 2 cm in diameter) which resolved completely without surgical intervention [33]. Melnik et al. reported that 4.4% of the patients (*n* = 7) treated with IORT had to be readmitted to hospital to receive intravenous antibiotics and drainage because of a wound infection, surgical debridement was not necessary in any of the cases. 11.4% of the patients (*n* = 18) developed minor wound complications that did not require readmission. They were treated with antibiotics, 7% (*n* = 11) of them needed additional drainage [47]. In the TARGIT A trial, 1.8% of the patients in the IORT group needed intravenous antibiotic therapy or surgical intervention due to a wound infection. A seroma needing more than three aspirations was observed in 2.1% of the patients in the IORT group [16]. In a single-arm prospective study by Senthi et al., 51% of the patients (*n* = 28) developed seroma after IORT treatment and 33% (*n* = 18) required at least one aspiration for symptomatic relief [48]. Zur et al. reported that 10.1% of the patients (*n* = 40) developed seroma after IORT, of whom 23 (57.5%) needed an aspiration [33]. Even though a few studies addressing complications after IORT exist, they mostly do not provide information on how these complications were handled, but are limited to a mere reporting of the types of complications with according prevalences [25, 28, 38, 49].

In our cohort, frequent findings at initial presentation were seroma, wound infection, and suture dehiscence (60% each). The majority of patients showed additional redness (80%). As initial treatment modalities, we applied antibiotic therapy administered for more than 3 days in eight cases (60%) and puncture of the seroma in three cases (30%). However, to manage the present severe complication lastingly, the patients of the presented cohort had to undergo multiple smaller and/or larger surgical interventions. Despite the low overall complication rate, in rare cases, extensive and disfiguring procedures such as mastectomy were necessary to resolve complications. While IORT constitutes a safe treatment option for eligible patients, our investigation delineates the minor, yet serious risk of developing a severe complication after receiving IORT that may require protracted and onerous treatment modalities. According to the literature, complications after IORT were treated by administration of antibiotics, surgical drainage, secondary suture or aspiration of seroma in the majority of cases. In our presented cohort we observed that in addition to a conservative treatment approach, surgical intervention was necessary. Even though in the majority of our patients smaller interventions such as excision of a necrotic area/fistula or secondary suture were sufficient to manage the complication, in some cases, greater interventions such as flap surgery or mastectomy were required for long-term control.

### Limitations

There are several limitations to our study that should be noted. Only a small number of patients from a single center were included, hence reducing generalizability as the complications observed may be influenced by surgical technique or postoperative care. Another shortcoming of the study is its retrospective design that may have introduced a selection bias. Due to the methodology (identifying the patients with major complications by chart review) there is no claim for completeness of cases as, for example, women might have consulted another physician outside of our hospital for treatment of wound complications.

## Conclusion

Even though IORT is a safe and efficient treatment method, we observed major complications that may require arduous and burdensome treatment in about 2.5% of the patients. This information should be part of the preoperative discussion with the patient. An increasing applicator size, a short tumor–skin distance, the omission of prophylactic antibiotic therapy, and a short time interval between IORT and the initiation of WBRT have been identified as possible risk factors for provoking major complications after IORT. In order to identify complications at an early stage, paying close attention to potential first signs of a complication during follow-up visits is recommended. In case a complication does not resolve with conservative management, smaller surgical interventions such as excision of a necrotic area/fistula or secondary suture might constitute a treatment option to consider. Greater interventions such as flap surgery or mastectomy are only necessary in a very small minority of cases.
